# Confirmatory factor analyses of the Mandarin Chinese version of the perceived stressors in intensive care units among healthcare professionals

**DOI:** 10.3389/fpubh.2025.1434440

**Published:** 2025-03-03

**Authors:** Yonge Gao, Xia Chen, Shuang Zhang, Jiawei Yang, Rui Liu, Wendy Moyle, Lihui Pu, Wei Shen

**Affiliations:** ^1^Nursing School, Shandong University of Traditional Chinese Medicine, Jinan, Shandong, China; ^2^Department of Neurology, Shandong Provincial Hospital Affiliated to Shandong First Medical University, Jinan, Shandong, China; ^3^School of Foreign Language, Shandong University of Traditional Chinese Medicine, Jinan, Shandong, China; ^4^Menzies Health Institute Queensland, Griffith University, Nathan Campus, Brisbane, QLD, Australia; ^5^School of Nursing and Midwifery, Griffith University, Nathan Campus, Brisbane, QLD, Australia; ^6^Department Internal Medicine, Section Nursing Science, Erasmus MC, University Medical Centre Rotterdam, Rotterdam, Netherlands

**Keywords:** intensive care units (ICU), perceived stressors in intensive care units (PS-ICU), Chinese, healthcare professionals (HCPs), psychometric properties

## Abstract

**Background:**

The Perceived Stressors in Intensive Care Units (PS-ICU) scale was designed to assess both general and occupational stressors experienced by healthcare professionals (HCPs) under normal circumstances. It has demonstrated good psychometric properties in three languages: French, Spanish, and Italian. The aim of the present study was to translate the scale into Mandarin Chinese and to examine its construct validity and reliability.

**Methods:**

This study was conducted from April 2022 to October 2023. In phase I, the scale was translated into Mandarin Chinese following the Cross-cultural adaptation guidelines and reviewed by expert panels. In phase II, the reliability and validation were tested by 530 HCPs working in tertiary grade A hospitals from two provinces (Shandong and Sichuan) in China. Fifty participants were contacted to evaluate the test–retest reliability and underwent a follow-up investigation 2 weeks after completing the initial online survey.

**Results:**

The content validity ratio for the 50 questions varied between 0.8 and 1, with every item having S-CVI values exceeding 0.92. After removing 4 items, the Exploratory Factor Analysis (EFA) results revealed six factors. Confirmatory Factor Analysis (CFA) affirmed construct homogeneity, comprising of (1) lack of fit with families and the organizational functioning, (2) emotional load associated with patient and family, (3) difficulties associated with teamwork, (4) issues associated with workload and human resource management, (5) issues associated with complex/at-risk situations and skill, (6) and suboptimal care situations. The comprehensive scale displayed strong internal consistency (the total Cronbach’s *α* = 0.96) and showed high 2-week test–retest reliability (Person’s *r* = 0.95). The Job Content Questionnaire (JCQ) was employed to assess the criterion-related validity, alongside with the Maslach Burnout Inventory-Hunman Services Survey (MBI-HSS), which revealed either positive or negative associations between PS-ICU and these measures.

**Conclusion:**

The final 46-item Mandarin Chinese version of the PS-ICU scale is reliable and valid for evaluating perceived stressors among HCPs under normal ICU conditions. It may significantly identify perceived stressors in the ICU providing a foundation for focused intervention research.

## 1 Introduction

The Intensive Care Unit (ICU) is a unit that provides centralized rescue and independent medical treatment for patients with life-threatening conditions. Working in an ICU that requires constant technological challenges, crisis decision-making in emergencies, and the burden of end-of-life care can be overwhelming for healthcare professionals (HCP), including physicians and nurses (1–3). These highly demanding experiences can lead to high work-related stress (4–6). Prolonged exposure to these factors is likely to bring with it a series of problems, such as anxiety, depression, burnout, and subsequent deterioration in service quality (7–10). Therefore, developing an accurate measure that evaluates the levels of perceived unique stressors among HCPs is essential to preserve and support their workplace health, as well as improve the quality of healthcare services.

The onset of the global COVID-19 pandemic further hastened the evolution of the critical care field towards high specialization and collaboration, exposing HCPs in ICU settings to new demands and challenges (11–13). Investigations suggested HCPs experienced high-stress levels from family and organizational functions, emotional burden from patients and their families, and ethical dilemmas (14–16). However, a systematic review (17) reported that among the 22 existing stress measures, such as the Intensive Care Unit Environmental Stressors Scale (ICUESS) (18) and the Intensive Care Unit Nurse Job Stressors Scale (19), few addressed family and organizational stressors, and none were designed specifically for HCPs. Choosing the most suitable tools with sufficient psychometric qualities from the existing measures is challenging.

The perceived stressors in intensive care units (PS-ICU) scale was developed by Laurent et al. (20). This scale includes two versions used to measure perceived stressors experienced by HCPs in the ICU. One version, with 50 items, covers six broad domains to explore general or specific stressors under normal conditions, while the other, containing 27 items, focuses on stressors encountered during a pandemic outbreak. Good psychometric properties have been evaluated in four countries: France, Canada, Spain, and Italy, and three languages, including French, Spanish, and Italian. This scale was translated and validated in China, exhibited cross-cultural solid validity when tested with Chinese samples (16, 21). Nevertheless, its application of PS-ICU, translated by Geng et al. (21), was limited to ICU nurses, with no indication of its use among ICU physicians. The criterion-related validity of the two translated tools remains unmeasured and unvalidated. Additionally, the correlation coefficient of some items remains low and the exploratory factor analysis results extracted seven common factors because of cultural differences between the countries of origin and use. Therefore, it is crucial to make additional adjustments to the scale’s items about cultural factors before advocating for its widespread adoption.

Our current study focused on HCPs in the ICU setting and aimed to investigate the psychometric properties of the PS-ICU. Specifically, in phase I, we aimed to translate the 50-item PS-ICU into Mandarin Chinese and successfully adapt cross-cultural adaptation comments from experts. In phase II, we evaluated the factor structure, convergent validity, criteria validity, internal consistency, and 2-week test–retest reliability of the Chinese PS-ICU among ICU HCPs. Our hypothesis suggested that the PS-ICU encompasses six comprehensive dimensions, addressing both general and occupational stressors inherent to the ICU, and exhibits good psychometric properties.

## 2 Methods

### 2.1 Phase I: the development of the PS-ICU scale Mandarin Chinese version

Ethical approval was received from the Ethics Committee of Shandong Provincial Hospital, Affiliated with Shandong First Medical University (SWYX: No. 2023–480). Following the Guideline for Translation and Cultural Adaptation for Health-related Quality of Life (22), we first obtained permission to translate and utilize the PS-ICU from the original author, Dr. Laurent Alexandra. The initial translation of the English version of PS-ICU into Chinese was performed by Zhang, a native Chinese speaker employed in the English sector, who is proficient in English translation but without familiarity with the scale. The translated draft was then sent to four experts, encompassing psychology, nursing, humanities, social sciences, and public health, all of whom were native Chinese speakers and proficient in English. Following the feedback, minor adjustments were made by the two authors. Subsequently, a master’s student with 4 years of study experience in an English-speaking country conducted the back-translation of the Mandarin Chinese PS-ICU into English. The original author of the PS-ICU confirmed the back-translated version as interchangeable with the original English version.

The content validity of the PS-ICU was evaluated by an expert panel convened from October 11, 2022, to March 30, 2023. This panel comprised of five physicians and nursing professors in ICU research recruited from the university or affiliated hospital. After recruitment, the panelists received a study information sheet via emails or WeChat, outlining the study’s purpose, risks and benefits, refinement process, contact details and the PS-ICU scale. Those who provided consent and returned their signed informed forms were then requested separately to evaluate the relevance of every item in two rounds of consultations. In each round, every item was evaluated on a scale of 1 to 4 points, where 1 represented highly irrelevant and 4 represented highly relevant. Ratings of 1 and 2 were grouped as irrelevant (assigned a value of 0), while scores of 3 and 4 were considered relevant (assigned a value of 1). Finally, discussions were held with five HCPs who volunteered to participate in the PS-ICU, and the results suggested that they thoroughly comprehended each item without any recommendations put forth in the pre-final version phase. A copy of the Chinese version of the PS-ICU is shown in Supplementary material.

### 2.2 Phase II: validity and reliability of the PS-ICU Mandarin Chinese version

#### 2.2.1 Sample

A purposeful and snowball sample of ICU physicians and nurses was recruited from two provinces (Shandong and Sichuan) in China from April 2023 to October 2023. With the subject-to-item ratio of 10:1, a sample size of 500 participants was needed as the Mandarin Chinese version of PS-ICU consists of 50 items (23). Furthermore, a sample size of at least 550 was recommended to allow for drop-out (24).

A total of 585 eligible physicians and nurses who had worked in the ICU of a tertiary grade A hospital for ≥1 year were enrolled, with 549 individuals completing online questionnaires via the survey platform.^1^ At the same time, the remaining participants opted for paper-based questionnaires. The survey link was distributed to individuals through WeChat, a widely used social media platform in China. Furthermore, 39 participants not affiliated with a tertiary grade A hospital and 16 participants who provided inconsistent responses were excluded from the analysis. The experts and the five volunteer HCP were not recruited in this Phase. Consequently, the analytical sample consisted of 530 participants. To assess test–retest reliability, 50 participants were invited to complete the survey again 2 weeks after their initial online survey.

#### 2.2.2 Measuring instruments

Participants were invited to fill out a demographics form with a series of self-report questionnaires. This study analysed data from the PS-ICU, Job Content Questionnaire (JCQ), and Maslach Burnout Inventory-Human Services Survey (MBI-HSS).

##### 2.2.2.1 Perceived stressors in intensive care unit (PS-ICU)

This is a self-reported scale used to measure general and occupational stressors among ICU healthcare professionals under normal circumstances, adapted from the original version by Laurent et al. (20). For each of the 50 items, participants respond to each item on a 5-point Likert scale bounded by 1 (never) and 5 (every day). The perceived stress score was calculated as the overall of all items. A higher score means a higher stress level.

##### 2.2.2.2 The job content questionnaire (JCQ)

This is a self-reported psychological scale utilized for assessing occupational stress, which Dr. Li Jian translated (25). It has 22 items distributed in three subscales: control (five items), job demands (nine items) and social support (eight items). Participants rate each item on a 4-point Likert scale bounded by 1 (strongly disagree) and 4 (strongly agree). Adding the scores from each domain yields the sum of its subscale scores, with higher scores indicating a greater degree of the measured domain. The Mandarin Chinese version of JCQ has showed good reliability and validity in healthcare workers working in northern hospitals in China (26). In this study, the Cronbach’s alpha coefficients were 0.74, 0.77, 0.86, 0.90 (total) respectively, showing good reliability.

##### 2.2.2.3 The Maslach burnout inventory-human services survey (MBI-HSS)

This is a 22-item self-report scale widely used to assess the occupational burnout of healthcare workers, adapted from the original version by Maslach and Jackson (27). It consists of three subscales: emotional exhaustion (EE), depersonalization (DP), and personal achievement (PA). Participants respond to each item on a 7-point Likert scale bounded by 0 (never) and 6 (every day). An elevated burnout level is characterized by an EE score of ≥27 or a DP score of ≥10. In the Chinese version, Cronbach’s *α* were 0.89, 0.79, and 0.80, respectively (28). In this sample, Cronbach’s α values were 0.69 for EE, 0.73 for DP, and o.82 for total. Only PA demonstrated limited internal consistency with a value of 0.40.

### 2.3 Data analysis

All data were analysed using SPSS 25.0. The significance level was established at 0.05. Data were expressed as categorical variables for number and frequency (percentage) or mean with standard error (SD) for continuous.

Content validity assesses whether the items in the instrument are relevant and comprehensive regarding the construct being measured using the Item-level Content Validity Index (I-CVI) and Scale-Level Content Validity Index (S-CVI). The greater the content validity score, the more precise the measurement of the target construct. The I-CVI for each item is calculated by dividing the number of experts who rated the item as relevant by the total number of experts. The S-CVI is calculated by averaging each item’s mean I-CVI and can be expressed as an S-CVI/Ave. An ICVI >0.79 (29) suggests that the item is relevant and does not require additional revision, while an S-CVI/Ave of 0.9 (30) indicates excellent content validity for the items.

The survey sample of 530 participants was randomly allocated into two groups of 265 to explore construct validity through exploratory factor analysis (EFA) and confirm it through confirmatory factor analysis (CFA). The EFA was executed through principal component analysis (PCA) and varimax rotation to establish a stable factor structure. Eigenvalues>1 were used to ascertain the number of final factors, while factor loading>0.4 was utilized to precisely define the dimensionality of items (31). PCA was conducted with criteria including a Kaiser-Meyer-Olkin (KMO) measure surpassing 0.06 and a statistically significant Bartlett test of sphericity (*p* < 0.05). Besides eigenvalue and scree plot, parallel analysis results were also used to determine the number of factors (32). We choose the 95th percentile, such as a significance threshold, to determine the cutoff point for significance.

The Mandarin Chinese PS-ICU item scores exhibited normal distribution characteristics, but the data did not demonstrate multivariate normality. Consequently, the robust maximum likelihood estimation method was used to estimate parameters. The fit of the CFA models was assessed using the ratio of chi-square value to degrees of freedom (χ^2^/df), root mean square error of approximation (RMSEA), Increasing Fitting Index (IFI), Tucker-Lewis fit index (TLI) and comparative fit index (CFI). As recommended by MacCallum et al. (33), models with χ^2^/df < 3.0, comparative fit index (CFI) ≥ 0.95, and root mean square error of approximation (RMSEA) ≤ 0.06 are considered to exhibit a good fit to the data, while the other indices such as IFI, TLI and CFI > 0.90, as well as RMSEA >0.07, can be construed as an acceptable fit.

Pearson correlations were computed between the PS-ICU subscales and measures of JCQ and MBI-HSS subscale to examine the validity of the Mandarin Chinese PS-ICU criteria. The interpretation of Pearson’s r correlation coefficients was as follows: weak (< 0.1); medium (0.3 < r < 0.5); strong (> 0.5) (34).

Internal consistency reliability was assessed utilizing Cronbach’s alpha coefficient, with a value of ≥0.8 considered indicative of good internal consistency and a value exceeding 0.9 considered excellent (35). As with internal consistency calculations, the test–retest correlations for the PS-ICU were analysed through Pearson’s r coefficient, which was computed based on the mean item score to assess consistency across repeated tests. The interpretation of Pearson’s r coefficients aligns closely with the previous explanation of Cronbach’s alpha coefficients.

## 3 Results

### 3.1 Content validity of the PS-ICU scale

In the first round of expert consultation, the internal validity test revealed an I-CVI of 0.6 for item 11, while I-CVI scores for the remaining items ranged from 0.8 to 1.0. The S-CVI/Ave was calculated as 0.976. In the subsequent round, all items garnered I-CVI scores falling within the 0.8 to 1.0 range, resulting in a computed S-CVI/Ave of 0.920. This implies that 92% of the items were deemed unambiguous, clear, and pertinent to the study participants. The outcomes regarding the content validity of the Chinese version of the PS-ICU are detailed in Table 1.

**Table 1:** The results of the two rounds of expert consultation.

Items	The number of experts rated as 3 or 4 points	I-CVI	Items	The number of experts rated as 3 or 4 points	I-CVI
	1st/2nd round	1st/2nd round		1st/2nd round	1st/2ndround
1	5/5	1.0/1.0	26	4/4	0.8/0.8
2	4/5	0.8/1.0	27	5/5	1.0/1.0
3	5/5	1.0/1.0	28	5/5	1.0/1.0
4	5/5	1.0/1.0	29	5/4	1.0/1.0
5	4/4	0.8/0.8	30	5/4	1.0/0.8
6	5/5	1.0/0.8	31	5/5	1.0/1.0
7	5/4	1.0/0.8	32	5/4	1.0/0.8
8	5/4	1.0/0.8	33	5/4	1.0/0.8
9	5/5	1.0/1.0	34	5/4	1.0/0.8
10	5/5	1.0/1.0	35	5/4	1.0/0.8
11	3/4	0.6/0.8	36	5/5	1.0/1.0
12	5/4	1.0/0.8	37	5/4	1.0/0.8
13	5/4	1.0/0.8	38	5/4	1.0/0.8
14	5/4	1.0/0.8	39	5/4	1.0/0.8
15	5/5	1.0/1.0	40	5/4	1.0/0.8
16	5/4	1.0/0.8	41	4/4	0.8/0.8
17	5/5	1.0/1.0	42	5/5	1.0/1.0
18	4/4	0.8/0.8	43	5/4	1.0/0.8
19	5/5	1.0/1.0	44	5/5	1.0/1.0
20	5/5	1.0/1.0	45	5/4	1.0/0.8
21	5/4	1.0/1.0	46	5/4	1.0/0.8
22	5/4	1.0/1.0	47	5/4	1.0/0.8
23	5/4	1.0/1.0	48	5/4	1.0/0.8
24	5/4	1.0/1.0	49	5/4	1.0/0.8
25	5/5	1.0/1.0	50	5/4	1.0/0.8

### 3.2 Validity and reliability of the PS-ICU scale

#### 3.2.1 Profile of participants

Of the 530 participants, 68.7% were females, 72.6% were nurses. The mean age and length of education were 31.6 years old (SD = 5.2) and 16.5 years (SD = 1.7). Thirty-six percent (*n* = 191) of the participants were single, while the rest were married; only 1 (0.2%) reported being divorced or separated. In this study, 66.5% of participants had a work duration exceeding 5 years, and 57.5% had over 5 years of experience in the ICU. Within this subset, individuals with a work history exceeding 10 years constituted 25.8%.

#### 3.2.2 EFA analysis

The results of the KMO (Kaiser-Meyer-Olkin) and Bartlett’s test of sphericity indicated that the data for the total PS-ICU score were appropriate for factor analysis (KMO = 0.924, Bartlett’s test χ2 (1035) =10688.17, *p* < 0.001). Utilizing PCA and varimax rotation as extraction methods, the initial analysis of scree plots revealed eight factors with eigenvalues exceeding 1, elucidating a total variance of 71.13%. Due to a double loading in item 29, the analysis identified seven factors following the removal of this item. However, the factor loading for item 10 had an absolute value of less than 0.4, leading to its exclusion. In addition, the seventh factor consisted of only two items (i.e., #18 and #39) and did not form a distinct dimension. Therefore, this factor was removed. Ultimately, six factors were obtained with a KMO value of 0.924 and a total variance of 70.302%. Examining the scree plot revealed the presence of six primary factors, followed by a sharp decline leading to the seventh factor. The parallel analysis results suggested that six factors were extracted (eigenvalues in descending order: 17.31, 4.78, 3.89, 3.02, 2.45, 2.11). So, we decided to select a 46-item scale with six factors. After applying maximum variance orthogonal rotation, the component matrix disclosed that all item loadings within their respective dimensions (0.486–0.848) surpassed the threshold of 0.40. Table 2 displays the factor structure and factor loadings of the PS-ICU.

**Table 2:** The PS-ICU scale items was loaded using principal axis exploratory factor analysis with Oblimin direct rotation (with the preliminary exclusion of items #10, #18, #29, #39.)

Items		Factor 1	Factor 2	Factor 3	Factor 4	Factor 5	Factor 6
6	Shortage of beds in the unit	0.85					
8	Inadequate or under-equipped healthcare space or defective materials	0.84					
26	Family whose beliefs or lifestyle are contradictory with my values or the functioning of the unit	0.84					
27	Family’s misunderstanding of the gravity of the diagnosis or the prognosis of the patient	0.84					
32	Lack of staff	0.80					
33	Non-supportive, aggressive or delirious patient	0.76					
41	Caring for a patient who should not be treated by the ICU	0.76					
34	Death of a patient with whom I had developed specialties	0.75					
35	Family which does not trust me or does not trust the team	0.75					
42	Uncertainty concerning the diagnosis or the therapy project of the patient	0.74					
7	Families’ distress or emotions		0.83				
28	Series of patient deaths in the unit over a short period		0.80				
31	Changes in the modalities of care or the therapy project depending on the doctor responsible for the patient		0.78				
50	Being on call or working nights		0.78				
37	Having to perform tasks for which I have neither knowledge nor skills		0.77				
43	Lack of respect for the patient (against his/her wishes, his/her integrity, his/her situation, etc.)		0.76				
49	Decision to stop or reduce treatment		0.76				
44	Patient suffering physically or psychologically		0.75				
45	Having to announce a bad diagnosis to the patient or his/her family or be present when such a diagnosis is announced		0.69				
46	Lack of equality in the distribution of tasks among healthcare professionals		0.66				
15	Lack of support from the administration			0.83			
13	Difficulty to find my place, have my skills recognized, or voice my opinion within the team			0.82			
17	Negative atmosphere prevailing in the team, gossip, rumours within the team			0.80			
21	Conflicts with members of the healthcare team			0.79			
14	Noisy environment			0.79			
12	Incomprehensible or unnecessary care relative to the patient’s situation			0.77			
38	Assessed or judged by the other members of the team			0.77			
9	Disagreement and/or lack of coordination with other units concerning a patient’s treatment			0.74			
11	Too many professionals around the patient in an emergency situation			0.49			
20	Working pace or working hours hardly compatible with family or social life				0.81		
23	Schedule changes, overtime				0.78		
24	Working while experiencing difficult personal events				0.78		
30	Powerlessness or incompetence in supporting families				0.72		
47	Unsuitable or under-equipped space to receive families				0.72		
36	Continuous and heavy workload				0.71		
48	Accumulated workloads resulting from clinical activity, training, research or teaching				0.70		
16	Risk of error, fear of doing a poor job					0.82	
25	Patient who deteriorates in an unexpected or unexplained manner					0.78	
22	Conflicts with members of the healthcare team					0.78	
40	Treating complex or serious pathologies					0.78	
19	Plaintive patient who makes many requests					0.77	
1	Socially isolated end-of-life patient or one with no immediate family						0.81
2	Colleague not doing his/her work properly						0.80
3	Lack of recognition (from the patient, the family, the team, the hierarchy)						0.78
4	Contradictory information given by other healthcare professionals to the family						0.78
5	Caring for young patients or who have young children						0.75

Factor 1 = lack of fit with families and the organizational functioning; Factor 2 = emotional load associated with patient and family; Factor 3 = difficulties associated with team working; Factor 4 = issues associated with workload and human resource management; Factor 5 = issues associated with complex/at-risk situations and skill; Factor 6 = suboptimal care situations.

The first factor comprised ten items (i.e., 6, 8, 26, 27, 32, 33, 34, 35, 41, 42) exhibiting high loadings, explaining 21.68% of the variance in the model (eigenvalue = 2.18). This factor was labeled “lack of fit with families and the organizational functioning.” The second factor, “emotional load associated with patient and family,” consisted of ten items (i.e., 7, 28, 31, 50, 37, 43, 49, 44, 45, 46). The third factor, “difficulties associated with teamwork,” included nine items (i.e., 15, 13, 17, 21, 14, 12, 38, 9, 11). The fourth factor, “issues associated with workload and human resource management,” comprised seven items (i.e., 20, 23, 24, 30, 47, 36, 48). The fifth factor, “issues associated with complex/at-risk situations and skill,” consisted of five items (i.e., 16, 25, 22, 40, 19). The sixth factor, “suboptimal care situations,” included five items (i.e., 1, 2, 3, 4, 5).

#### 3.2.3 CFA analysis

**Figure 1:**
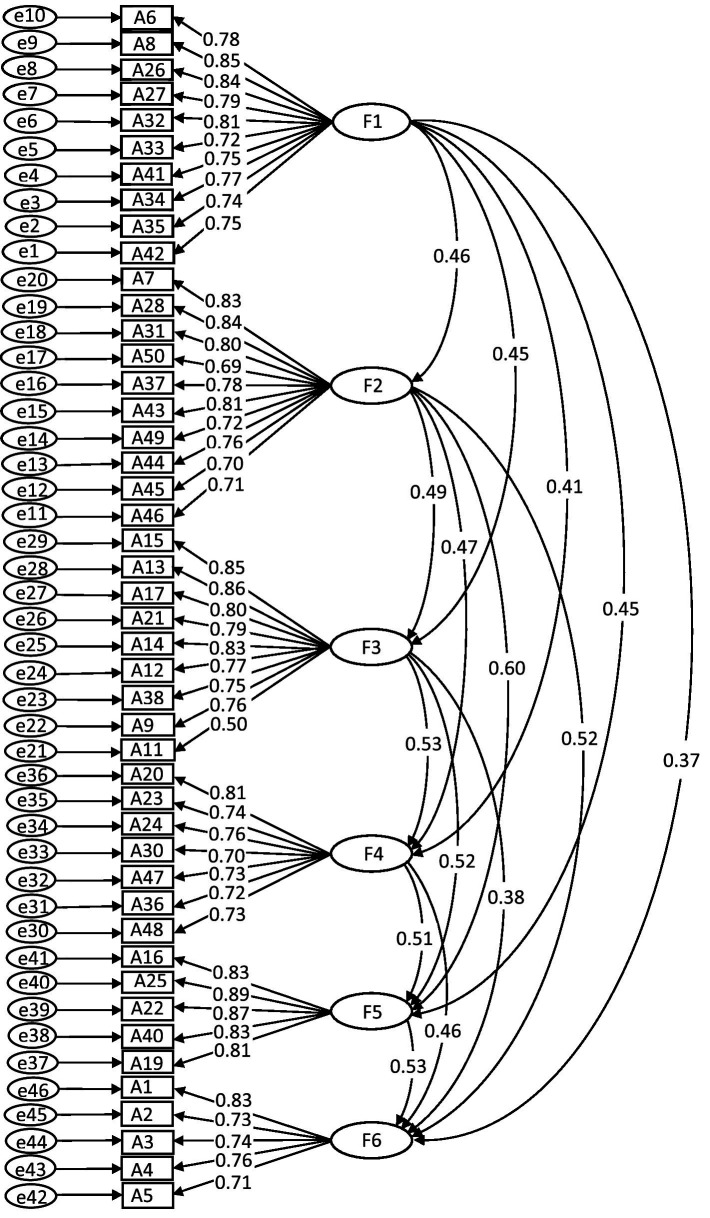
Graphical representation of six-factor model with factor loadings.

According to the adaptation criteria suggested by MacCallum et al. (33), the 6-factor solution provided an acceptable fit to the data, χ2/df = 1.778, robust RMSEA = 0.054, TLI = 0.907, IFI = 0.913, robust CFI = 0.912. A graphic representation of the revised model of six-factor loadings is shown in Figure 1.

#### 3.2.4 Criterion-related validity

In general, all the Mandarin Chinese PS-ICU subscales were significantly and positively correlated with job demands and negatively correlated with control and social support of JCQ. The correlations ranged between 0.12 and 0.60, which were moderate to strong in size, indicating satisfactory convergent validity. Overall, emotional exhaustion scores exhibited significant and positive associations with all the Mandarin Chinese PS-ICU subscales, while personal accomplishment scores showed a significant negative association. Additionally, all subscales positively correlated with personal accomplishment scores, with only Factor 1, Factor 2, and Factor 3 showing statistical significance. These findings are reported in Table 3.

**Table 3:** The correlations between the Mandarin Chinese PS-ICU and MBI-HSS.

	Factor 1	Factor2	Factor3	Factor4	Factor 5	Factor 6
Convergent validity (correlations with the JCQ)						
Job demands	0.16^**^	0.29^**^	0.17^**^	0.15^**^	0.25^**^	0.57^**^
Control	−0.42^**^	−0.30^**^	−0.46^**^	−0.23^**^	−0.39^**^	−0.27^**^
Social support	−0.17^**^	−0.13^*^	−0.22^**^	−0.19^**^	−0.21^**^	−0.12^*^
Concurrent validity (correlations with the MBI-HSS)						
Emotional exhaustion	0.19^**^	0.29^**^	0.17^**^	0.21^**^	0.37^**^	0.37^**^
Depersonalization	0.12^*^	0.13^*^	0.25^**^	0.09	0.11	−0.03
Personal accomplishment	−0.22^**^	−0.30^**^	−0.30^**^	−0.23^**^	−0.27^**^	−0.29^**^

^*^p<0.05, ^**^p<0.01; JCQ, Job Content Questionnaire; MBI-HSS, Maslach Burnout Inventory-Hunman Services Survey.

#### 3.2.5 Reliability

The internal consistency, assessed through Cronbach’s alphas, ranged from 0.88 to 0.94 for factors 1 to 6 and was 0.96 for the overall PS-ICU score. The 2-week test–retest reliability ranged from 0.84 to 0.96 for the six subscales and 0.95 for the total scale.

## 4 Discussion

This study translated the PS-ICU (20) into Mandarin Chinese and evaluated its psychometric properties, specifically focusing on the validity and reliability of its application. Our combined results support the psychometric soundness, grounded in the demonstrated internal consistency and stability (over 2 weeks) and their respective scores’ content and convergent validity for the final Chinese version of the 46-item PS-ICU.

As for the content validity, two rounds of expert correspondence were undertaken. Despite achieving an S-CVI/Ave of 0.976 in the first round, the I-CVI for Item 11 was only 0.6, indicating results falling below the desired threshold. This item and other items were modified as they were considered crucial aspects of perceived stressors based on expert opinions. Therefore, a follow-up consultation round was conducted. Ultimately, the instrument had excellent content validity, as evidenced by I-CVI ranging from 0.8 to 1 and S-CVI/Ave of 0.920. However, some items experienced a decline in I-CVI, and the S-CVI/Ave decreased in the second round compared to the first. In early December 2022, the first round of inquiries coincided with a notable increase in the daily recording of COVID-19 cases in China (36). The impact extended beyond patients, affecting HCPs and their families with widespread infections. This presented substantial physical and psychological challenges for the healthcare workforce, ultimately constraining the experts’ capacity to conduct thorough analyses. Consequently, the depth of the feedback provided in the first round was insufficient. The second round was conducted in March 2023 during a period of decreased infections within the hospital. In this context, experts could provide more thorough and unbiased assessments. As a result, there was a decrease in I-CVI for some items and S-CVI/Ave. Additionally, all items maintained I-CVI > 0.79 (29) and S-CVI/Ave > 0.9 in the second round (37), indicating that the consultation process could be concluded.

According to Tate (35), a value of 0.80 or higher indicates ideal internal consistency. Accordingly, the internal consistency reliabilities for the Mandarin Chinese PS-ICU subscales were deemed ideal (0.86–0.93), with the total scale’s Cronbach’s alphas of 0.96. These findings support the internal consistency reliability of the Mandarin Chinese PS-ICU. The six subscales also showed good 2-week test–retest reliability ranging from 0.84 to 0.96. This favorable stability reinforced that the Chinese scale version is a reliable tool.

The high KMO value of 0.924 and the significant result of Bartlett’s sphericity test supports the data appropriateness for factor analysis. The extraction of six factors is consistent with both scree plot analysis and parallel analysis results, indicating robustness in the factor structure. Like the 6-factor structure observed in the original PS-ICU (20), the Mandarin Chinese PS-ICU exhibited a 6-factor structure after deleting four items (Items 10, 18, 29 and 39). Item deletion informed by EFA results or modification indices from CFA is a routine practice in scale development and validation of scales. Moreover, most of our participants were recruited from university-affiliated hospitals, where a considerable proportion of patients were critically ill, including a notable number of young patients. These hospitals have strict regulations that apply to the staff (38). Therefore, some deleted items (ie., items 5 and 15) in the Hu et al. research (16) remained in this study. The final Chinese version of PS-ICU was a 46-item scale with 6-factors: (1) lack of fit with families and the organizational functioning (10 items); (2) emotional load associated with patient and family (10 items); (3) difficulties associated with teamwork (9 items); (4) issues associated with workload and human resource management (7 items); (5) issues associated with complex/at-risk situations and skill (5 items); and (6) suboptimal care situations (5 items). In the EFA procedure, it was observed that certain items did not correspond with the dimensions as categorized in the original version of the scale. For example, four items (items 8, 32, 33 and 34) were grouped under Factor 1, whereas another 4 items (items 9, 11, 12 and 15) were attributed to Factor 3. Item 50 “Being on call or working nights” loaded higher on Factor 2 (0.78) than Factor 4, while it loaded higher on Factor 4 (0.52) in the original scale than Factor 1. In the current study, two items (items 19 and 22) have been reclassified to Factor 5, previously assigned to Factor 6; conversely, item 3 has been reassigned to Factor 6, formerly categorized under Factor 5. A similar factor structure and some factor scores were reported by Geng et al. (21) in a sample of Chinese nurses with a 48-item version of PS-ICU. Above all, no cross-loadings were observed for each item in the current factor analysis. As a result, their respective categorizations were retained. It is not clear, however, whether this is due to Chinese culture or the working environment and extends to expressions related to perceived stress (39). Further research is necessary to address this question.

The findings from criteria-related validity analysis revealed a correlation between PS-ICU and JCQ, as well as MBI-HSS. The correlation was relatively satisfactory for JCQ, indicating that the Chinese scale version (33) maintains an equivalent level of convergence as the original PS-ICU. Indeed, this scale captured some different aspects perceived by HCPs working in the ICU. Similarly, previous studies showed a positive correlation between MBI-EE and MBI-DE with PS-ICU (20, 40, 41). However, MBI-PA exhibited a negative correlation with PS-ICU. Therefore, this exploration of concurrent validity for the PS-ICU scale demonstrated satisfactory results with MBI-HSS, which was used to measure occupational burnout as an external criterion.

## 5 Limitations and future research

Recognizing the limitations of the current findings is crucial. Initially, we employed a purposeful and snowball sampling method to recruit ICU physicians and nurses affiliated with tertiary Grade A hospitals from two provinces in China to assess the psychometric properties of the Mandarin Chinese PS-ICU. Consequently, the generalizability of the findings to other groups may be limited, for example, samples from lower-grade hospitals. Second, the MBI-HSS scale demonstrated good reliability and validity in previous studies, but the reliability of some subscales (i.e., DP) in the current study is not entirely satisfactory. Third, the final 46-item Mandarin Chinese version of PS-ICU still has too many items, especially for clinical practitioners. As such, a more concise tool should be validated, considering the increasing demand for psychological instruments focused on emotional response and management among healthcare professionals in the ICU.

## 6 Conclusion

In summary, the results of this study indicated that the Mandarin Chinese version of the PS-ICU scale had good reliability and validity, making it a valuable tool for evaluating perceived stressors among physicians and nurses in ICU settings. The Mandarin Chinese PS-ICU showed associations with control, job demands, social support, and the occupational burnout of HCPs. Employing the PS-ICU in future research will enhance our comprehension of general and special stress concerns among Chinese-speaking HCPs in the ICU.

## Data Availability

The raw data supporting the conclusions of this article will be made available by the authors, without undue reservation.

## Footnotes

^1^ https://www.wjx.cn/
