# Supplementary material for: Confirmatory factor analyses of the Mandarin Chinese version of the perceived stressors in intensive care units among healthcare professionals
Source: Front Public Health. 2025 Mar 3;13:1434440. doi: 10.3389/fpubh.2025.1434440 (PMC11911360; doi:10.3389/fpubh.2025.1434440)
Supplement: Supplementary file 1 [file Data_Sheet_1.pdf]

Supplementary Material

中文版 ICU 医护人员感知压力源量表

以下是重症监护病房内可能遇到的一些情况。对于每种情况，请指出你是否在你的病房内经历过这些特定的情况。如果您有经历过的话，请选择遇到该情况时您的压力水平（1 分~54 分）。其中，1 分代表从未经历过，5 分代表压力极大。

题目	从未经历过	没有任何压力	有一点压力	压力相当大	压力极大
1. 照护与社会隔离的临终患者或没有直系亲属的患者	1	2	3	4	5
2. 同事没有妥当地完成当班工作，导致护理/治疗缺乏连贯性	1	2	3	4	5
3. 缺乏认同感,如来自患者、家属、团队及上级的认同感	1	2	3	4	5
4. 与其他医务人员向家属提供的信息相矛盾	1	2	3	4	5
5. 照护病情重且预后差的年轻患者或有年幼子女的患者	1	2	3	4	5
6. 病房内床位不足	1	2	3	4	5
7. 面对患者家属痛苦或期待的表情	1	2	3	4	5
8. 医疗空间不足、设备缺乏，或耗材量大而无法满足患者需求	1	2	3	4	5
9. 关于患者治疗，与其他科室医护人员有分歧和/或缺乏配合	1	2	3	4	5
10. 抢救时，患者周围聚集太多医护人员而干扰治疗	1	2	3	4	5
11. 对患者进行医护人员难以理解或不必要的护理，如家属强烈要求的护理操作	1	2	3	4	5
12. 在团队中难以找到自我位置、表达自我观点（如治疗理念被同事完全否决），或自我技能难以得到认可	1	2	3	4	5
13. 环境嘈杂	1	2	3	4	5
14. 缺乏来自管理层的支持	1	2	3	4	5
15. 存在出错的风险，担心做不好工作	1	2	3	4	5
16. 团队内消极氛围弥漫，存在闲言碎语甚至流言蜚语	1	2	3	4	5
17. 面对提出过多无理要求的患者及其家属	1	2	3	4	5
18. 工作节奏和工作时间很难与家庭及社会生活相协调	1	2	3	4	5
19. 与同事起冲突	1	2	3	4	5
20. 与患者无法顺利的沟通	1	2	3	4	5

21.计划有改变，如不在计划范围之内加班/非听班时间段内加班/下班后接到科室与工作有关电话等	1	2	3	4	5
22.经历个人困难事件（如生理期、身体不舒服、家人生病需要照顾）时仍需工作	1	2	3	4	5
23.面对患者病情变化难以预料或无法解释	1	2	3	4	5
24.患者家属存在一些与自己的价值观不符或与病房工作模式相矛盾的理念或生活方式	1	2	3	4	5
25.关于疾病诊断，患者家属对其严重性或预后存在误解	1	2	3	4	5
26.病房里接受治疗的患者短期内相继去世	1	2	3	4	5
27.无能为力或无法胜任照顾自己的家庭	1	2	3	4	5
28.关于护理模式或治疗方案，其变化取决于主管医生	1	2	3	4	5
29.缺少人手	1	2	3	4	5
30.面对不合作、有攻击性或谵妄的患者	1	2	3	4	5
31.与我建立了特别情感关系的患者去世	1	2	3	4	5
32.患者家属不相信我或团队的专业性	1	2	3	4	5
33.持续而繁重的工作量	1	2	3	4	5
34.必须完成自己没有掌握技能的任务	1	2	3	4	5
35.被团队其他成员或领导评价	1	2	3	4	5
36.处理复杂或严重疾病	1	2	3	4	5
37.治疗或照护非 ICU 准入指征的患者	1	2	3	4	5
38.患者的诊断或治疗方案存在不确定性	1	2	3	4	5
39.对患者缺乏尊重，如违背其意愿、处境等进行治疗或护理操作	1	2	3	4	5
40.面对身体或心理上遭受痛苦的患者	1	2	3	4	5
41.不得不向患者或家属宣布不好的诊断，或宣布诊断时必须在场	1	2	3	4	5
42.医疗专业人员之间任务分配不合理，如奖金分配、工作安排、学习进修等	1	2	3	4	5
43.在不适合或设施简陋的房间接待患者家属，如在装有录像的谈话间与患者家属沟通	1	2	3	4	5
44.因参加临床活动、培训、研究或教学而累积的工作负担	1	2	3	4	5
45.做出停止/减少治疗或照护的决定	1	2	3	4	5
46.随时待命或值夜班	1	2	3	4	5
